# Nursing for the Insured

**Published:** 1922-12

**Authors:** W. Gill Hodgson

**Affiliations:** Clerk Liverpool Insurance Committee


*To the Editor of* THE HOSPITAL AND HEALTH REVIEW.

### Nursing for the Insured.

*To the Editor of* THE HOSPITAL AND HEALTH REVIEW.

SIR,—I observe from page 14 of your last issue that you propose to refer to the subject of Nursing for the Insured in your next number, as the result of a letter appearing in the November issue from the Clerk to the Cheshire Insurance Committee. I think it may be of interest if the experience of another insurance area is made available. Home nursing has been advocated for a very long time, and until recently has been looked upon as an adjunct of medical benefit ; but when additional benefits under the Insurance Acts were being arranged out of the surpluses shown by the valuation of Society funds an effort was made by Societies to keep the arrangement and control of this and other benefits within their own hands, without the intervention of Insurance Committees set up primarily for the administration of medical benefit and other benefits in the nature of medical benefit.

Since any additional benefit must be made available to all the members of the Society, and there was not a Nursing Service available in all parts of the kingdom nor any central organisation therefor, the Queen's Jubilee Institute for Nurses, who had been approached by certain Approved Societies, undertook the organisation of the matter, and obtained the affiliation of most of the local Nursing Associations. But even so, there is still a considerable part of the country in which there is no nursing service provided, as witness page 19 of your last issue, wherein it is stated that "a complete service for rural areas would require 1,400 more associations." As is well known, the Queen's Jubilee Institute is not a "Nursing Association," and has merely induced local nursing associations to affiliate to it for the purpose of providing the necessary central organisation demanded by the larger Approved Societies desirous of granting nursing as an additional benefit.

Because of the absence of such a central organisation and the further fact that "nursing service was not available in all parts of the kingdom," many Societies took the line of least resistance, and supplied the additional benefits by means of an increase to the cash benefits. But nursing may be provided under Section 21 of the Act of 1911, in which case the cost is charged to the Benefits Fund of Societies, for all its members, as well as under the scheme of additional benefits in cases in which surplus funds are available. The latter can only be provided for those members entitled to additional benefits under a scheme requiring the approval of the Ministry of Health, while under the former the service may be provided for every existing member, and the specific consent of the Ministry is not necessary. If, therefore, nursing be regarded as essential, it should be available to every member, and this can best be done under Section 21.

In my view, the Insurance Committee organisation should also be utilised to secure a nursing service for the whole kingdom. The whole country is divided into Insurance Committee areas, and such Committees could easily arrange with existing Associations to provide the necessary service, or to encourage the creation of new Associations within its area, where needed—chiefly county areas. The Cheshire Committee states that the result of its inquiries has not been sufficiently satisfactory to justify the Insurance Committee in proceeding to put the scheme into operation in its area. The question which at once arises is at what point may a Committee be said to be justified ?

I have always held that a scheme should be provided and put into operation in every area, no matter how inadequately Approved Societies may support it, if only for the purpose of providing the necessary service for the small (chiefly local) Societies. This was the view held in Liverpool which led to the appointment of a Nursing Committee, comprising representatives of those Approved Societies taking part in the scheme (and forming the majority of the Committee), Insurance Committee, Panel Committee, City Council and local Nursing Associations. The Nursing Committee has representation also on the local Nursing Association, and thus co-ordination is effected. Conferences were arranged with all Societies having members locally, and letters were addressed to them urging participation in the scheme ; but the response from purely local Societies has been disappointing, and leads one to think that either there is not the strong desire for nursing which is stated, or that Societies having earmarked their surplus funds for additional benefits hesitate to incur expenditure under Section 21 of the principal Act on the assumption that such expenditure compensated by a reduction in the amount now payable in sickness and disablement benefit.

The arrangements made by certain Approved Societies through the Queen's Jubilee Institute and those made on behalf of other Societies by the Local Nursing Committee are working concurrently in Liverpool, and every effort is made to bring to the notice of doctors, insured persons and others the services available, whether made by this Committee or otherwise. Clearly, "the only real satisfactory solution of this nursing problem is the setting up of a comprehensive scheme for the whole of the country." In these days, when economy is the paramount consideration, any such comprehensive scheme is not within the realm of practical politics. But that is no reason why attempts should not be made to solve the problem, on however small a scale, in order that the advantage of experience may be derived. It is for this reason principally that a scheme has been inaugurated and is being continued here ; and it is hoped that every Committee will take the matter up for the benefit of the local Societies, even though the large Societies as yet decline to take part therein.

I am, Sir,
Yours faithfully,
W. Gill Hodgson,
Clerk Liverpool Insurance Committee.

